# Supplementary material for: Role and mechanism of NCAPD3 in promoting malignant behaviors in gastric cancer
Source: Front Pharmacol. 2024 Apr 22;15:1341039. doi: 10.3389/fphar.2024.1341039 (PMC11070777; doi:10.3389/fphar.2024.1341039)
Supplement: Supplementary file 11 [file DataSheet2.ZIP › GSEA/Oncogenic signature/my_analysis.Gsea.1599461962066/gsea_report_for_WT_1599461962066.html]

Report for WT 1599461962066 [GSEA]

| GS  follow link to MSigDB | GS DETAILS | SIZE | ES | NES | NOM p-val | FDR q-val | FWER p-val | RANK AT MAX | LEADING EDGE || 1 | ESC\_J1\_UP\_LATE.V1\_DN | Details ... | 26 | 0.37 | 1.75 | 0.021 | 0.223 | 0.580 | 675 | tags=77%, list=48%, signal=145% |
| 2 | MYC\_UP.V1\_UP | Details ... | 15 | 0.46 | 1.73 | 0.016 | 0.128 | 0.634 | 593 | tags=80%, list=42%, signal=137% |
| 3 | PRC2\_EED\_UP.V1\_DN | Details ... | 15 | 0.44 | 1.69 | 0.024 | 0.109 | 0.743 | 698 | tags=93%, list=49%, signal=183% |
| 4 | GCNP\_SHH\_UP\_LATE.V1\_DN | Details ... | 16 | 0.38 | 1.52 | 0.069 | 0.204 | 0.962 | 601 | tags=75%, list=43%, signal=129% |
| 5 | CRX\_DN.V1\_DN | Details ... | 15 | 0.31 | 1.18 | 0.247 | 0.678 | 1.000 | 42 | tags=20%, list=3%, signal=20% |
| 6 | STK33\_DN | Details ... | 34 | 0.22 | 1.11 | 0.317 | 0.703 | 1.000 | 130 | tags=21%, list=9%, signal=22% |
| 7 | EIF4E\_DN | Details ... | 15 | 0.29 | 1.09 | 0.349 | 0.651 | 1.000 | 286 | tags=40%, list=20%, signal=50% |
| 8 | PIGF\_UP.V1\_UP | Details ... | 27 | 0.23 | 1.05 | 0.405 | 0.646 | 1.000 | 205 | tags=26%, list=15%, signal=30% |
| 9 | RPS14\_DN.V1\_DN | Details ... | 27 | 0.21 | 0.98 | 0.497 | 0.695 | 1.000 | 17 | tags=11%, list=1%, signal=11% |
| 10 | PRC1\_BMI\_UP.V1\_UP | Details ... | 16 | 0.20 | 0.80 | 0.706 | 0.927 | 1.000 | 14 | tags=13%, list=1%, signal=12% |
| 11 | CYCLIN\_D1\_UP.V1\_DN | Details ... | 24 | 0.15 | 0.67 | 0.859 | 1.000 | 1.000 | 544 | tags=46%, list=39%, signal=73% |
| 12 | ERBB2\_UP.V1\_DN | Details ... | 25 | 0.13 | 0.61 | 0.937 | 0.993 | 1.000 | 101 | tags=12%, list=7%, signal=13% |
| 13 | MEK\_UP.V1\_DN | Details ... | 27 | 0.11 | 0.54 | 0.957 | 0.961 | 1.000 | 35 | tags=7%, list=2%, signal=7% |
Table: Gene sets enriched in phenotype **WT (3 samples) **[plain text format]****
